# Co-targeting of delta-like ligand 4 (DLL4) and vascular endothelial growth factor a (VEGF) with programmed death 1 (PD1) blockade inhibits tumor growth and facilitates anti-tumor immune responses

**DOI:** 10.1186/2051-1426-3-S2-P373

**Published:** 2015-11-04

**Authors:** Minu Srivastava, Christopher Murriel, Rui Yun, Erin Mayes, Hyun-Bae Jie, Fumiko Axelrod, Ming-Hong Xie, Trevor Bentley, Belinda Cancilla, Raymond Tam, Gilbert O'Young, Ann Kapoun, John Lewicki, Tim Hoey, Austin Gurney, Park Angie Inkyung

**Affiliations:** 1 Oncomed Pharmaceuticals, Redwood City, CA, USA

## 

Blocking DLL4, a Notch ligand, effectively inhibits tumor growth by increasing non-functional angiogenesis and decreasing the cancer stem cell (CSC) population. Preclinical studies have demonstrated inhibition of tumor growth by anti-DLL4 treatment and have led us to enter demcizumab, an anti-DLL4 mAb, into ongoing clinical trials. Vascular endothelial growth factor A (VEGF A) also plays a central role in inducing tumor angiogenesis. VEGF signaling is also involved in recruiting immune suppressive myeloid cells. Therefore, targeting VEGF could induce favorable immune responses against cancer. We developed a bispecific monoclonal antibody that blocks both DLL4 and VEGF which is in Phase I clinical trials. In the present study we compare the impact of anti-DLL4 in combination with anti-VEGF and anti-DLL4 in combination with anti-VEGF and anti-Programmed Cell Death Protein 1 (PD1) on anti-tumor immune responses. Our data demonstrate that the triple blockade of DLL4-VEGF-PD1 significantly inhibited tumor growth with more pronounced tumor regression. Anti-DLL4 treatment reduced IL17 production, an effect not observed with anti-PD1, blockade of DLL4-VEGF or DLL4-VEGF-PD1, suggesting that blocking DLL4 alone and together with VEGF or VEGF and PD1 might have different mechanisms for regulating immune responses. Anti-PD1 increased specific CD8^+^ T cell-mediated IFN-gamma production while decreasing IL6. Interestingly, IL2 was increased at the tumor site by blockade of DLL4-VEGF-PD1 compared to controls. Since IL2 is required for secondary population expansion of CD8^+^ memory T cells, increased IL2 in the triple combination group suggests potential for increased T cell activation, maintenance and memory T cell function, as compared to single agent anti-DLL4 and anti-PD1. Memory CD8^+^ T cell frequencies were increased within the total CD8^+^ T cell population by DLL4-VEGF-PD1 triple blockade. Therefore, these results show that co-targeting of DLL4 and VEGF with PD1 might be an effective and durable anti-cancer therapy in part by promoting anti-tumor immune responses and inhibiting pro-tumor immune responses.

